# Ocular Rosacea: Don’t Forget Eyelids and Skin in the Assessment of This Stubborn Ocular Surface Disease

**DOI:** 10.7759/cureus.51439

**Published:** 2024-01-01

**Authors:** Joobin Khadamy

**Affiliations:** 1 Ophthalmology, University Hospital of Umeå, Umeå, SWE

**Keywords:** speed dry eye questionnaire, ocular surface disease index (osdi), the national rosacea society, systemic treatment for rosacea, eyelid care for ocular rosacea, eyelid hygiene for blepharitis, azithromycin in rosacea, azithromycin in mgd, ocular rosacea, acne rosacea

## Abstract

Ocular rosacea, a subset of rosacea affecting the ocular surface, poses a diagnostic challenge due to its elusive presentation and overlapping symptoms with other ocular surface diseases (OSDs). This report emphasizes the critical role of a comprehensive evaluation, particularly focusing on eyelid and skin assessment, in diagnosing and effectively managing ocular rosacea-related ocular surface symptoms. The case study highlights a 69-year-old female initially diagnosed with common dry eye disease, subsequently identified with ocular rosacea following a meticulous examination revealing subtle ocular and skin manifestations. Treatment encompassed a tailored approach combining systemic and local therapies, emphasizing proper eyelid hygiene. Objective improvements were observed in ocular surface parameters and patient-reported symptom scores, showcasing the significance of an integrated approach addressing ocular and dermatological aspects in managing ocular rosacea. This report underscores the importance of heightened clinical suspicion, thorough assessments, and comprehensive management strategies in optimizing outcomes for patients with OSD, particularly ocular rosacea.

## Introduction

The causes of ocular surface symptoms span various factors like environmental, inflammatory, and mechanical elements [1]. In the assessment checklist for patients with ocular surface disease (OSD), an integral step before commencing the slit lamp examination involves evaluating the skin, eyelids, and blinking behavior. The creation of a checklist, akin to a pilot's pre-flight routine, serves as a valuable tool for ophthalmologists, preventing oversights in diagnosis and treatment. The significance of eyelids and skin health in ocular surface health is often neglected. Their role in dry eye and associated conditions is highlighted in blepharitis and meibomian gland dysfunction (MGD) [2].

The potential link between blepharitis or MGD and ocular rosacea is likely often overlooked [3], which affects skin, eyelids, and eyes. Ocular rosacea management requires a holistic treatment approach [3,4]. This report illustrates an undiagnosed ocular rosacea case, showing marked improvements after a tailored treatment addressing rosacea and eyelid-related issues.

## Case presentation

A 69-year-old female patient was referred to the eye clinic by her primary care physician because of a longstanding issue of redness and eye irritation, with suspicion of Sjögren's disease. The patient had experienced periods of exacerbation in symptoms and red eye, for which she had been prescribed topical antibiotics due to suspicion of bacterial conjunctivitis. There was no prior record of the patient having visited the eye clinic. Additionally, the patient had been using artificial tears and lubricant gels regularly but had not experienced the desired improvement in her condition.

During the initial assessment conducted at the eye clinic by a junior ophthalmology resident physician, dry eye disease was suspected in the patient. This suspicion arose due to the patient having a borderline tear break-up time (TBUT=9 seconds), despite a normal Schirmer test I result. Consequently, the patient was prescribed a three-week course of mild preservative-free corticosteroid eye drops. Unfortunately, there was minimal to no subjective or objective improvement following this treatment.

**Figure 1:**
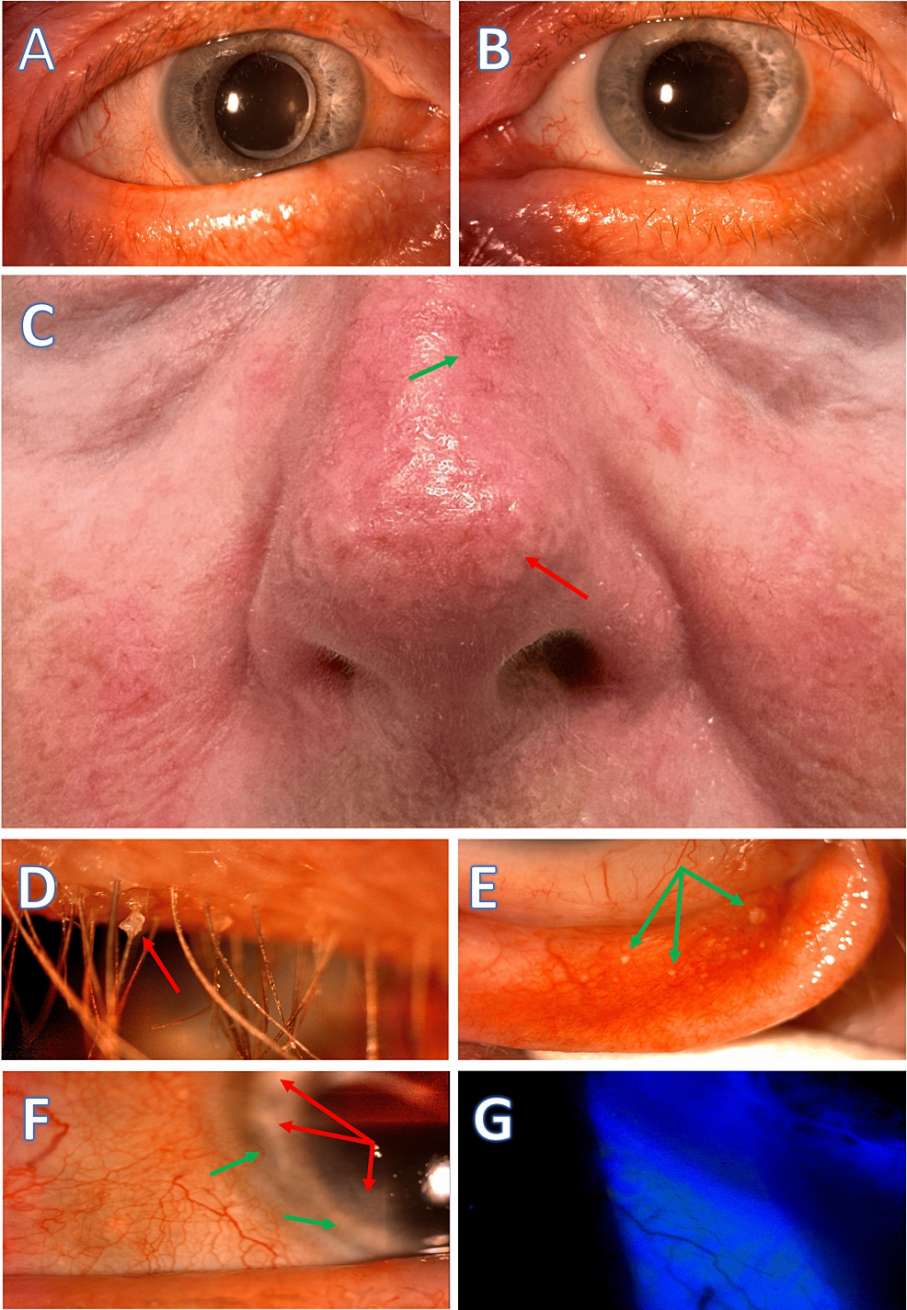
Skin and ocular findings in a patient with rosacea before treatment. A and B: Rounded, notched, thickened, and telangiectatic eyelid margins. C: Phymatous rosacea affecting the nose (rhinophyma) and cheeks, characterized by skin thickening, irregular surface nodules, telangiectasia (green arrow), fibrosis, and increased sebaceous gland volume (red arrow). D: Anterior blepharitis with waxy collarettes (red arrow) at the base of the eyelashes. E: Conjunctival concretions (green arrows) on the palpebral conjunctiva, are indicative of chronic inflammation. F: Ingrowth of abnormal blood vessels (green arrows), and scarring in the peripheral cornea (red arrows) suggesting possible prior episodes of marginal keratitis. G: Staining of the bulbar conjunctiva in the lower areas near the eyelid margins.

During a subsequent follow-up visit by a senior physician, several concerning findings were observed in the patient's eye examination. These included the presence of collarets at the base of the eyelashes, thickening, and irregularity of the eyelid margins, the presence of concretion cysts in the palpebral conjunctiva, limbal pannus formation, and signs suggesting potential previous episodes of marginal keratitis. Furthermore, the patient exhibited telangiectasia on the malar area and nose. These clinical observations concluded that the patient's symptoms were likely attributed to ocular rosacea with blepharitis (Figure 1).

The patient received guidance on minimizing aggravating factors, which included recommendations to avoid spicy foods, alcohol, prolonged exposure to sunlight, and excessive heat, as well as specific cosmetics and soaps. Management of the condition involved adhering to a regular eyelid hygiene routine using eyelid wet tissues and warm eye compresses. To further facilitate understanding, the clinic provided a handout that visually depicted the correct technique.

Additionally, the patient experienced localized palpable discomfort at the location of a sizable concretion cyst, which was subsequently incised and drained. To address potential photosensitivity during the summer season and better tolerance, an alternative oral antibiotic (azithromycin) was prescribed for the patient's rosacea treatment.

The severity of ocular surface disease was evaluated using the Ocular Surface Disease Index (OSDI) [5] and vital dye staining. Before treatment, the ocular staining score, determined through Lissamine green staining of the bulbar conjunctiva, was 2, but it decreased to 0 in the three-month follow-up. The corneal fluorescein staining pattern score was already 0 before treatment, indicating the absence of punctate epithelial erosions (PEEs).

The patient's subjective experience, including the frequency and severity of symptoms, was assessed using the Standard Patient Evaluation of Eye Dryness (SPEED) Questionnaire [5], which decreased from an initial score of 16 to 7 out of 28. Additionally, the OSDI score decreased from 23 (indicating moderate severity in the range of 23-32) to 15 (indicating mild severity in the range of 13-22) out of 100.

## Discussion

Rosacea, affecting around 10% of the population, primarily targets middle-aged women with fair complexions, blue eyes, and blonde hair. Nearly half of those with rosacea suffer symptoms related to eye discomfort, dryness, and sensitivity to light [3,4]. A subtype of this condition, ocular rosacea, often poses a diagnostic challenge due to its manifestation with nonspecific complaints, causing difficulties in accurate diagnosis by eye specialists or dermatologists [3,4]. Recognizing its distinctive features becomes critical to prevent underdiagnosing common ocular symptoms associated with rosacea [6].

The Global ROSacea COnsensus Panel (ROSCO) highlights that even with minimal skin involvement, ocular rosacea can be identified by specific features like lid margin abnormalities, corneal issues, or scleral inflammation [6]. It frequently appears as blepharitis, often accompanied by symptoms like gritty eyes, irritation, dryness, and light sensitivity. Severe cases might lead to complications such as corneal ulcers, impacting vision. Distinguishing ocular rosacea from other OSDs requires a comprehensive evaluation involving various diagnostic measures, although the current guidelines for the assessment of OSD may not sufficiently emphasize assessing eyelid and skin health [4]. Hence, clinicians should reconsider evaluation protocols, considering ocular rosacea as a potential diagnosis in cases of ocular surface symptoms, even with minimal skin involvement [6].

For an effective diagnosis of ocular rosacea in patients presenting with ocular symptoms, a systematic approach involving various tests is recommended. In a stepwise approach toward OSD and ocular rosacea, the assessment of patients starts with a complete history taking and asking about the patient's subjective experience through questionnaires like OSDI. This aids in establishing a diagnosis and grading of symptoms, validating the medical necessity for further intervention and stage-wise treatment selection, and assessing the subjective effectiveness of treatments [4,6].

Our patient had moderate ocular surface symptoms score while she had just mild findings on tear film tests. This aligns with prior research indicating that ocular rosacea can significantly affect both the structure and function of the meibomian glands, even in cases where tear film tests reveal mild abnormalities while patients exhibit elevated scores for ocular surface symptoms [7].

The patient's skin should be meticulously examined, paying particular attention to the forehead, nose, chin, and cheeks to identify any visual abnormalities. Both eyes should be assessed for potential bilateral involvement, recognizing that symmetry may not always be present. Lid margin signs, including rounding or notching of the lid margins, vascularity like telangiectasia, and the presence of collarettes around the eyelashes, should be evaluated. Investigation of MGD should involve an expressibility assessment to quantify meibum quality and expressibility grade, following the guidelines set by the International Workshop of MGD [8].

A thorough examination of the conjunctiva, covering both tarsal and bulbar regions, with a special focus on the inferior region, should be conducted. A detailed evaluation of the cornea should be performed, searching for signs such as corneal vascularization, peripheral thinning, or corneal infiltrates, especially in the inferior peripheral cornea. Employing fluorescein staining of the cornea and lissamine green staining of the conjunctiva is essential for measuring the TBUT to assess lipid layer instability. Grading of fluorescein staining should be carried out on both the cornea and two conjunctival zones immediately after TBUT measurement, utilizing a grading system such as the Oxford Grading System [9].

By following this systematic diagnostic approach, healthcare professionals can effectively diagnose ocular rosacea in patients presenting with ocular symptoms, ensuring a comprehensive evaluation of potential signs and factors associated with the condition.

Effective management of OSD and ocular rosacea hinges on two crucial elements: setting realistic patient expectations to ensure ongoing commitment to treatment and employing combination therapies to address the multifaceted factors contributing to the condition. Individualized treatment plans should consider various aspects, including symptoms, trigger factors, patient preferences, psychological aspects, and specific needs [10].

Ocular rosacea, which tends to be a persistent condition, requires patients to understand the long-term nature of their treatment. Complete cure is rare, and control is the primary goal, recognizing that some patients may experience structural changes or require an extended recovery period. Patient education about rosacea and suitable treatments plays a pivotal role in treatment success [10].

While supplemental lubrication is key for mild to moderate OSD, ocular rosacea management primarily focuses on proper lid hygiene beyond the use of preservative-free artificial lubricants. Patients with OSD are often inclined to immediately pursue medical therapies without receiving sufficient emphasis or guidance regarding proper lid hygiene. Emphasizing and instructing patients on lid hygiene is essential, often aided by illustrated handouts to enhance compliance. Effective OSD management necessitates a strong emphasis on proper lid hygiene and comprehensive assessments to address lid irregularities and promote tear film stability. Combining clinical therapies to address various symptoms simultaneously can yield the best outcomes [3].

The enthusiasm surrounding meibomian gland rehabilitation as a potential breakthrough in dry eye therapy is evident from the numerous innovative methods being introduced for gland rehabilitation. It's worth noting that even patients without obvious MGD but experiencing significant dry eye symptoms may exhibit an increased presence of inflammatory cells in the proximal tissue and conjunctiva. This suggests that some dry eye symptoms might originate from the palpebral conjunctiva of the eyelids [3].

Meibomian gland rehabilitation is promising for dry eye therapy. Effective office and at-home treatments for meibomian gland health and ocular surface inflammation are likely to yield only limited and short-term benefits unless underlying eyelid problems associated with OSD are addressed first [11].

Avoiding exacerbating factors, such as spicy foods, alcohol, sunlight exposure, heat, and specific cosmetics and soaps, can help reduce exacerbations. For ocular rosacea exacerbations, topical antibiotics, with or without steroids, are effective. In severe dry eye cases, cyclosporine A eye drops may be necessary [3,6].

In moderate to severe cases, oral doxycycline is the first-choice therapy, and other options like tetracycline, azithromycin, and metronidazole can be considered for all severities. Topical ivermectin cream has shown promise due to its dual action against inflammation and Demodex [3,6].

Surgical treatments like amniotic membrane transplantation or keratoplasty may be considered in rare cases of non-healing or deep corneal ulcers at high risk of perforation [3,6].

The frequency of follow-up care should align with the severity of signs and symptoms. The treatment can be optimized by a comprehensive patient history review conducted at least annually, coupled with an updated list of available interventions [3,6].

The presented patient demonstrated a positive response to a combination of local and systemic therapies, complemented by education on the importance of eyelid hygiene. The limitations of the current report may include the absence of a control group for comparative analysis, potential biases due to the single-case study design, and the lack of long-term follow-up to assess sustained treatment effects or relapse rates in ocular rosacea management.

## Conclusions

When dealing with patients experiencing ocular surface symptoms, especially those with unresponsive cases, it's essential to adopt a systematic approach using a checklist. The examination of such patients should commence with an assessment of their skin condition, eyelid health, and blinking patterns. Particular attention should be given to identifying signs of rosacea in individuals displaying ocular surface signs and symptoms.

Utilizing quantitative measurements through questionnaires and grading of ocular findings facilitates a stepwise, tailored treatment approach and helps in evaluating treatment effectiveness during follow-up appointments. It is imperative to educate patients about the significance of proper eyelid hygiene and provide a realistic prognosis for rosacea, emphasizing that it can be managed but not fully cured.

Patients should also be informed about the potential impact of dietary modifications and advised on avoiding factors that can exacerbate their condition. Additionally, the use of oral antibiotics can significantly alleviate symptoms and improve objective rosacea findings in patients.
